# How Monocytes Contribute to Increased Risk of Atherosclerosis in Virologically-Suppressed HIV-Positive Individuals Receiving Combination Antiretroviral Therapy

**DOI:** 10.3389/fimmu.2019.01378

**Published:** 2019-06-19

**Authors:** Anthony Jaworowski, Anna C. Hearps, Thomas A. Angelovich, Jennifer F. Hoy

**Affiliations:** ^1^School of Health and Biomedical Sciences, RMIT University, Bundoora, VIC, Australia; ^2^Department of Infectious Diseases, Alfred Hospital and Monash University, Melbourne, VIC, Australia; ^3^Life Sciences Discipline, Burnet Institute, Melbourne, VIC, Australia

**Keywords:** HIV, monocytes/macrophages, atherosclerosis, foam cells/macrophages, inflammation

## Abstract

Combination antiretroviral therapy (ART) is effective at suppressing HIV viremia to achieve persistently undetectable levels in peripheral blood in the majority of individuals with access and ability to maintain adherence to treatment. However, evidence suggests that ART is less effective at eliminating HIV-associated inflammation and innate immune activation. To the extent that residual inflammation and immune activation persist, virologically suppressed people living with HIV (PLWH) may have increased risk of inflammatory co-morbidities, and adjunctive therapies may need to be considered to reduce HIV-related inflammation and fully restore the health of virologically suppressed HIV+ individuals. Cardiovascular disease (CVD) is the single leading cause of death in the developed world and is becoming more important in PLWH with access to ART. Arterial disease due to atherosclerosis, leading to acute myocardial infarction (AMI) and stroke, is a major component of CVD. Atherosclerosis is an inflammatory disease, and epidemiological comparisons of atherosclerosis and AMI show a higher prevalence and suggest a greater risk in PLWH compared to the general population. The reasons for greater prevalence of CVD in PLWH can be broadly grouped into four categories: (a) the higher prevalence of traditional risk factors e.g., smoking and hypertension (b) dyslipidemia (also a traditional risk factor) caused by off-target effects of ART drugs (c) HIV-related inflammation and immune activation and (d) other undefined HIV-related factors. Management strategies aimed at reducing the impact of traditional risk factors in PLWH are similar to those for the general population and their effectiveness is currently being evaluated. Together with improvements in ART regimens and guidelines for treatment, and a greater awareness of its impact on CVD, the HIV-related risk of AMI and stroke is decreasing but remains elevated compared to the general community. Monocytes are key effector cells which initiate the formation of atherosclerotic plaques by migrating into the intima of coronary arteries and accumulating as foam cells full of lipid droplets. This review considers the specific role of monocytes as effector cells in atherosclerosis which progresses to AMI and stroke, and explores mechanisms by which HIV may promote an atherogenic phenotype and function independent of traditional risk factors. Altered monocyte function may represent a distinct HIV-related factor which increases risk of CVD in PLWH.

## Introduction

For PLWH at the present time, HIV infection is associated with different outcomes depending on access to care and treatment. In resource-constrained settings, those without access to antiretroviral therapy (ART), or with limited access to expensive, alternative ART that is required when first-line therapies fail, experience progressive disease with decreased CD4+ T cell counts ultimately leading to death from AIDS–related diseases. For PLWH who have access to effective antiretroviral regimens, current evidence suggests that life expectancy approaches that of HIV-negative individuals in the general population. Whilst the risk of AIDS and AIDS-related mortality may be low, long term health outcomes for these individuals depend on how soon after infection they are treated, with the best outcomes predicted for those who present early in infection with relatively preserved CD4 counts (1–5). Large cohort studies of persons from the United States, and from North America and Western Europe, have shown that the gap in life expectancy between PLWH and HIV-negative individuals has narrowed as a function of the year in which ART was initiated (2, 6). This is likely due to use of more potent and tolerable ART regimens and adoption of treatment guidelines recommending earlier commencement of ART at diagnosis of HIV.

## Cardiovascular Disease in PLWH

Despite a reduction in AIDS-related mortality, HIV infection remains associated with an increased risk of age-related, inflammatory diseases which cause significant morbidity such as non-AIDS cancers, neurocognitive impairment and cardiovascular disease (CVD). Cardiovascular diseases, or diseases of the heart and vasculature are major causes of morbidity and mortality. They include ischaemic coronary artery disease (chronic angina and AMI), cerebrovascular disease (stroke and transient ischaemic attack), other heart diseases (including arrhythmias, inflammatory heart disease, valvular disease, rheumatic heart disease, and heart failure) and other cardiovascular diseases (peripheral vascular disease, aortic aneurysms, and hypertensive disease). The incidence of many of these conditions is increased in PLWH [for recent reviews see (7, 8)]. The incidence of CVD may be decreased following ART and the impact of ART will depend on the degree to which inflammation and immune activation are decreased following HIV virologic suppression. This review focuses on coronary artery disease (CAD) in well-resourced settings since it is the major cause of mortality in the general population and is an increasing cause of mortality in PLWH as the median age of this population is increasing. Specifically, we look at mechanisms that increase the risk of sub-clinical atherosclerosis which is elevated in these populations and leads to the major causes of death from CVD, i.e., AMI and ischaemic stroke, and focus on the role of activated monocytes as a likely effector contributing to these mechanisms.

## Elevated Risk of CAD in virologically suppressed PLWH

In virologically suppressed individuals on stable and effective ART, CAD has become one of the leading causes of death (9). Early hospital record-based studies that compared the rate of AMI in large cohorts of HIV-positive and HIV-negative individuals presenting at the same US hospitals between 1996 and 2004, showed that the relative risk of AMI was 1.75-fold higher after adjusting for age, gender (*sic*), race, diabetes, hypertension, and dyslipidemia (10). Similarly, a study of US veterans enrolled since 2003 and followed-up until 2009, confirmed that the incidence of AMI was higher [HR = 1.48 after adjustment for Framingham risk score (FRS), co-morbidities and substance abuse] in HIV-positive veterans matched demographically to HIV-negative veterans (11). In both of these studies, participants were not restricted to those virologically suppressed by ART, and information on the proportion of successful virological suppression were not presented. Similar results were obtained in analyses of incidence of AMI in the French Hospital Database on HIV (FHDH-ANRS CO4) cohort (12) where standardized mortality ratios were 1.4 [95% confidence: 1.3–1.6] and 2.7 [1.8–3.9] compared to the general population for men and women, respectively. More recent studies have reported that the incidence is also higher when restricted to type 1 events, i.e., those associated with atherosclerotic plaque rupture or thrombosis, which are relevant as inflammatory co-morbidities in PLWH who are virologically suppressed on ART (13).

The higher rate of AMI reported in the above studies contrasts with a gradual decline of incidence in individuals enrolled via the Kaiser-Permanente health plan (California, USA) as a function of when HIV patients started ART. In those individuals who commenced ART between 2010 and 2011, the rate of AMI decreased to the point where the incidence was not statistically different from that in HIV-negative controls (14) and also no difference was observed in rates of AMI in PLWH who commenced ART at CD4 cell counts of ≥500/μL (15). Other studies report a reduction in AMI incidence over time as well; Masia et al reported a decrease in standardized incidence rates in PLWH relative to the general population in Spain when the intervals between 2006–2009 and 2010–2014 were compared (16). The observed reduction in AMI risk may be, in part, due to prescription of ART regimens with lower associations with CVD, adoption of guidelines for earlier initiation of ART, and increased awareness of traditional risk factors for CVD in PLWH. The more frequent interactions of PLWH within healthcare settings, and the consequent impact on CVD risk monitoring and treatment, is difficult to correct for in cohort analyses, and may be an important confounder in comparing CVD risk in HIV-positive and HIV-negative populations. Also, we have limited long term data on CVD risk in virologically-suppressed PLWH and the HIV-related CVD risk in virologically suppressed PLWH on ART for long periods of time remains to be established.

## Why is CVD Risk Elevated in Virologically Suppressed HIV-Positive Individuals?

There are several potential and recognized factors that may elevate risk of CVD in PLWH.

### Traditional Risk Factors

A higher prevalence of traditional risk factors for CVD such as smoking (17–19), diabetes (20, 21), dyslipidemia (including hypercholesterolemia, triglyceridemia, low HDL cholesterol and abnormal fat distribution) and hypertension (22, 23) is found in HIV-positive populations in well-resourced settings. As many of these factors are modifiable, reduction in CVD risk attributable to traditional risk factors is as effective in PLWH as strategies and treatments for the general population (24). Recently, Althoff and colleagues have used data from the North American AIDS Cohort Collaboration on Research and Design to estimate the contribution of population-attributable risk factors for AMI, in addition to other non-AIDS comorbidities, and concluded that considerable reduction in risk could be obtained by reducing total cholesterol levels and hypertension in PLWH (25).

### Antiretroviral Drugs

The risk of AMI associated with ART use was robustly reported in the Data collection on adverse events of ARV Drugs (D.A.D) study (26) in which duration of ART was associated with an adjusted relative risk of AMI of 1.26 [1.12–1.41] per year. Specific drugs, or classes of drug, have also been associated with CV events; recent, but not cumulative use, of abacavir (27) and continued use of protease inhibitors (26, 28) including some recently-introduced combinations (29). The study by Ryom and colleagues using data from the D:A:D cohort, showed that cumulative use of the boosted protease inhibitor combination darunavir/ritonavir was associated with a 60% increase in baseline risk of CVD (using a composite endpoint that included AMI and stroke, and also sudden cardiac deaths and invasive cardiovascular procedures) over 5 years of use. In contrast, use of atazanavir/ritonavir was not associated with an increase in risk. These findings were not explained when analyses were controlled for effects of dyslipidemia or a protective effect of hyperbilirubinemia. Deleterious effects of antiretroviral drugs contribute to traditional CVD risk factors including increased circulating levels of cholesterol and triglycerides (26), visceral adiposity (30), reduced high-density lipoprotein-associated cholesterol (HDL_c_) levels (31), hypertension (32, 33) and metabolic syndrome (34–36) shown by studies linking the prevalence of these syndromes with years of ART (33). The hypothesized mechanism for the effect of current use of abacavir on AMI is via increased platelet reactivity, which reverses when abacavir is ceased (37, 38). Antiretroviral drugs probably mediate much of their effect on hypertension via their effects on other components of the metabolic syndrome such as diabetes, changes in HDL and low-density lipoprotein (LDL) levels and alterations in fat distribution which are themselves associated with hypertension; for a more complete discussion see (23, 33). Risks associated with ART can be ameliorated by avoidance of abacavir and specific protease inhibitors. Another factor is the effect that weight gain following ART initiation with various PI-based, integrase inhibitor-based and earlier non-thymidine analog-based regimens may have on CAD risk (39, 40). For further information, the reader is referred to a recent comprehensive review (41).

### HIV-Related Chronic Inflammation

Atherosclerosis is an inflammatory disease (42). A recent meta-analysis has confirmed an association of plasma markers of inflammation with CVD in PLWH (43) particularly interleukin 6 (IL-6), D-dimer and high-sensitivity C-reactive protein (hs-CRP), which are the most extensively studied biomarkers. However, in individual studies measuring various outcomes related to CVD, biomarkers of myeloid activation such as sCD14 and sCD163 have been more closely associated [(44–46) and discussed further below].

Monocytes/macrophages possess a full complement of pattern recognition receptors that promote inflammation by stimulating production of high levels of pro-inflammatory factors. Measuring their activation status, most conveniently via soluble plasma markers or cell surface markers on monocytes, is therefore a valuable approach to assessing inflammation and innate immune activation in individuals. Using cross sectional studies we reported that virologically suppressed PLWH have higher levels of circulating plasma biomarkers of inflammation and of myeloid activation compared to age-matched HIV-negative individuals, but similar to levels found in much older individuals (47, 48) which is consistent with findings from other laboratories (45, 49). We further estimated that the increase in immune activation was equivalent to an additional 2–4 years of aging in virologically suppressed individuals (50) and reasoned that this increased the risk of age-related inflammatory co-morbidities by a commensurate rate. We measured phenotypic markers on blood monocytes and reported their continued alteration in virologically suppressed individuals (47), however plasma biomarkers of monocyte/macrophage activation such as CXCL-10 correlate with monocyte subset and phenotypic alterations (51) and are a more convenient and robust measure in clinical studies.

The significance with respect to CAD of the extent to which immune activation and inflammation persist in PLWH on effective ART is evident from studies showing that measures of myeloid activation and inflammation are associated with non-calcified plaque (52, 53), coronary artery calcium (54, 55), carotid intima-media thickness (46) and predict cardiovascular events and death (56–59). Expression of tissue factor on monocytes, important for initiating platelet activation, is increased by TLR ligands such as LPS elevated in settings of HIV infection (60), although not at the concentrations found in the plasma of PLWH (61), and by thrombin (61). Schechter and colleagues further showed that tissue factor expression was restricted to CCR2+ classical monocytes (a discussion of human monocyte subsets is given below) although others have implicated CD16+ monocytes (62). While a discussion of late events in atherosclerosis and mechanisms of plaque rupture are outside the scope of this review, it is important to note that in a primate model of HIV, administration of an inhibitor of tissue factor activity on monocytes, Ixolaris, decreased immune activation (61). The association of monocyte activation markers and soluble myeloid activation biomarkers with incidence of CAD and with cardiovascular events is strong circumstantial evidence for a mechanistic role of activated monocytes in these processes. However, the mechanisms by which monocytes may contribute to CAD are best identified by functional comparisons of these cells present in PLWH and in well-matched HIV-negative individuals.

### HIV-Specific Mechanisms

HIV produces pathogenic factors such as the Negative Regulatory Factor, Nef, that are potential mediators of HIV-related morbidities. HIV-infected foam cell macrophages have been detected in coronary arteries obtained post-mortem from HIV-positive individuals, and Nef has been shown to stimulate their formation by inhibiting reverse cholesterol efflux from macrophages (63), suggesting a direct mechanistic link. The significance of these observations is discussed in more detail below.

## HIV-Associated Atherosclerosis

AMI follows thickening of the arterial wall by atherosclerotic plaque formation and eventual occlusion of coronary arteries. Ischaemic strokes result when unstable atherosclerotic plaques rupture and lodge in the brain, while peripheral artery disease shares similar initial events to CAD of plaque formation and narrowing of arteries at other sites. Both are significant co-morbidities elevated in PLWH. Atherosclerotic plaques identified in virologically suppressed PLWH are more likely to be non-calcified plaques considered to be unstable and high risk (64, 65).

As atherosclerosis underlies coronary artery diseases it is important to understand whether it is more prevalent in virologically suppressed HIV-positive individuals and how HIV impacts its development. Early studies reported increased pre-clinical atherosclerosis assessed by surrogate measures such as carotid artery intima media thickness (cIMT) in PLWH compared to HIV-negative controls (66, 67). More recently, Leon et al. reported that in a well-controlled virologically suppressed HIV-positive population with a low cardiovascular risk, as measured using the FRS, of <10%, 21% had sub-clinical atherosclerosis determined by carotid artery ultrasonography (68). In this group of patients, who had median FRS of 1% and all with an FRS <8%, traditional risk factors did not account for the high prevalence of sub-clinical atherosclerosis, but the authors found high IL-6 levels (>6.6 pg/mL) resulted in an odds ratio of 9 for sub-clinical atherosclerosis. These findings suggest an involvement of inflammation in heightened CVD risk in these subjects rather than the traditional risk factors accounted for by FRS (age, smoking, dyslipidemia, total and HDL cholesterol, systolic blood pressure).

An important factor underlying increased immune activation in PLWH is that most are cytomegalovirus (CMV) seropositive, and have an impaired ability to control CMV reactivation. CMV infection in PLWH has been associated with expansion of a unique population of adaptive natural killer cells which serve as a sensitive marker for the presence of CMV infection (69, 70). Using small cross-sectional studies of 93 PLWH and 37 healthy, HIV-negative controls, it has been shown that sub-clinical atherosclerosis (as measured by cIMT) is correlated with CD8 T cell responses to CMV antigen (pp65) (66). CMV infection in PLWH is also associated with expansion of CD8+ T cells expressing fractalkine receptor (CX3CR1; involved in endothelial homing and adhesion) and the PAR-1 receptor which can be activated by thrombin, although the impact of these cells on clinical atherosclerosis is unknown (71). Other studies have found correlations between CMV antibody (IgG) levels and carotid artery stiffness in HIV-positive women, although association of IgG with the prevalence of cardiovascular lesions was restricted to those who achieved virologic suppression (72). More recently, in a small cross-sectional study of 105 PLWH matched to 105 healthy, age- sex- and smoking-matched controls, a correlation was observed between CMV IgG levels and cIMT (73).

There are few well-controlled studies comparing the relative prevalence of atherosclerosis in well-managed virologically suppressed PLWH to appropriate HIV-negative controls. One such study found that the prevalence of coronary plaque as measured by Coronary Computed Tomography Angiography (CCTA) was higher in 78 HIV-positive men (of whom 95% were currently receiving cART and 81% were virologically suppressed) compared to 32 HIV-negative men recruited from the same clinics in Boston (59 vs. 34%, *p* = 0.02) (74). A similar larger study from the Multicentre AIDS Cohort Study confirmed these findings with a 1.25-fold higher prevalence of plaque after adjustment for cardiovascular risk factors (75). In contrast, studies from the Swiss HIV cohort have reported little or no increase in prevalence of high risk, non-calcified plaque and lower coronary atherosclerosis involvement than HIV-negative persons with a similar FRS (76). A smaller study demonstrated that HIV-positive women are more likely to have non-calcified plaque compared to HIV-negative women, after controlling for known cardiovascular risk factors, although prevalence of calcified plaque was not increased (52). The presence of non-calcified plaque is associated with higher levels of the macrophage activation marker sCD163 and with CVD in HIV-positive individuals (52, 53). A meta-analysis of 9 studies involving 1,229 HIV-positive individuals and 1,029 HIV-negative controls, concluded there was an increased prevalence of non-calcified plaque in PLWH as detected by CT (OR = 3.26 [1.30–8.18]) (77). Larger studies are required to measure the relative prevalence of non-calcified plaque in PLWH who commence ART with well-preserved CD4 counts.

## Accumulation of Activated Macrophages in the Ascending Aortic Arch is Associated with CVD in HIV-Positive Individuals

[^18^F] FDG-PET is a technique which uses positron emission tomography to image uptake of [^18^F] 2-deoxyglucose into cells and tissues. There is evidence [for a summary, refer to papers listed in (44)], and it is assumed, that the main cell type that accumulates [^18^F] 2-deoxyglucose is the activated monocyte-derived macrophage, and that therefore the intensity of radioactivity detected by PET reflects accumulation and activation of monocytes to the activated endothelium. More specific macrophage tracers are being developed which leverage the fact that macrophages and dendritic cells express CD206, the mannose fucose-receptor: these include ^99m^Tc-diethylenetriaminepentaacetic acid—mannosyldextran (Tilmanocept or LymphoSeek™) (78, 79) and radiolabeled anti-MFR monoclonal antibodies (80) but they have yet to be used extensively in studies of CVD risk in HIV.

In a proof-of-concept study, a greater uptake of [^18^F] FDG occurred in both the carotid arteries and the aorta of 9 PLWH compared to 5 HIV-negative participants (81). Similarly, in 27 virologically suppressed HIV-positive individuals without evidence of cardiovascular disease and age-, sex-, and CVD risk-matched to 27-HIV-negative individuals, the HIV-positive participants had greater accumulation of ^18^F radiolabel in the ascending aortic arch at levels comparable to those measured in 27 HIV-negative individuals with cardiovascular disease (44). These results provide evidence that HIV infection, even with successful virologic suppression, is associated with similar levels of arterial inflammation as found in individuals with pre-existing CVD. Follow-up studies from the same group showed that the arterial inflammation in a similar group of 41 HIV-positive patients was associated with higher numbers of high-risk coronary plaque (82).

The accumulation of [^18^F] FDG is evidence for activated macrophages accumulating in the arteries of the heart, and its association with plaque with low attenuation and positive remodeling emphasizes the clinical significance of this accumulation, but it does not provide a mechanism by which this occurs. In HIV-positive participants, higher accumulation of FDG was moderately associated with elevated plasma levels of sCD163, but not D-dimer or hs-CRP (44). This is consistent with a specific link with activated macrophages but not with generalized inflammation or thrombotic disease. Unfortunately, plasma markers of endothelial activation were not investigated. Taken together these studies suggest that atherosclerosis is elevated in PLWH receiving effective ART due to a greater accumulation of monocyte-derived macrophages in coronary arteries leading to formation of non-calcified, high risk plaques. Significantly, it has been reported that whereas treatment of PLWH with atorvastatin improved lipid profiles and reduced the prevalence of non-calcified plaque in these individuals after 12 months, there was no reduction in the accumulation of FDG measured by [^18^F] FDG-PET (83). This suggests that accumulation of activated monocyte-derived macrophages in the heart is caused by factors other than dyslipidemia and that it may represent an independent risk factor for CVD in PLWH. This may be specifically associated with myeloid cell activation. It is therefore important to understand how effective therapies used to reduce hypercholesterolemia are at reducing inflammation and monocyte activation that may contribute to CVD in PLWH. Initial reports from the SATURN-HIV trial showed that whereas treatment with the statin rosuvastatin was effective at reducing LDLc levels in HIV patients after 24 weeks, it was not effective in reducing inflammatory markers studied including well-established markers such as IL-6, hsCRP, and soluble TNF receptor isoforms (84). In contrast, lipoprotein-associated phospholipase A2 [an enzyme produced by myeloid cells and an independent predictor of CVD (85)] was significantly decreased in the statin arm compared to the placebo arm of the trial. Follow up studies of the same cohort indicated greater reduction in immune activation as assessed by various biomarkers: after 48 weeks, treatment with rosuvastatin reduced levels of the inflammatory marker IP-10 (interferon-γ-inducible protein-10 i.e., CXCL10) and the myeloid activation marker sCD14, as well as the proportion of non-classical monocytes expressing tissue factor (86). However, even after 48 weeks treatment with rosuvastatin, inflammatory markers IL-6, hsCRP, D-dimer and soluble TNF receptors were not reduced in this study (*ibid*). These data underscore the importance of monocyte activation in the mechanism leading to increased atherosclerosis and emphasizes the necessity of examining the functional properties of monocytes in these individuals to understand how their altered behavior might favor atherosclerotic plaque formation.

## Initiation of Atherosclerotic Plaque Formation

Atherosclerotic plaques tend to form in arteries at sites of perturbed blood flow such as near bifurcations in arteries. The initiation of atherosclerotic plaque formation is accelerated by the presence of an activated endothelium at these sites. In individuals with traditional risk factors such as high levels of triglycerides, total cholesterol, or LDL_c_ in plasma, lipid accumulates in the intimal layer beneath the endothelium where it is oxidized by reactive oxygen intermediates produced by activated endothelial cells and macrophages. The action of oxidative pathways derived from endothelial cells and macrophages produce different oxidized species in LDL particles (87, 88) but both lipid species have been found in atherosclerotic plaque-derived lipids, suggesting that both endothelial cells and macrophages participate in this pathway (89). Oxidized LDL (oxLDL) is a known risk factor for CVD which has been shown in one study of HIV-positive individuals (91% of whom received ART) (90) and in a second study (of patients with varying degrees of HIV viremia) (91) to be elevated in plasma, and is associated with subclinical atherosclerosis in this population (92, 93).

## The Role of Monocyte Subsets in Atherosclerotic Plaque Formation

**Figure 1:**
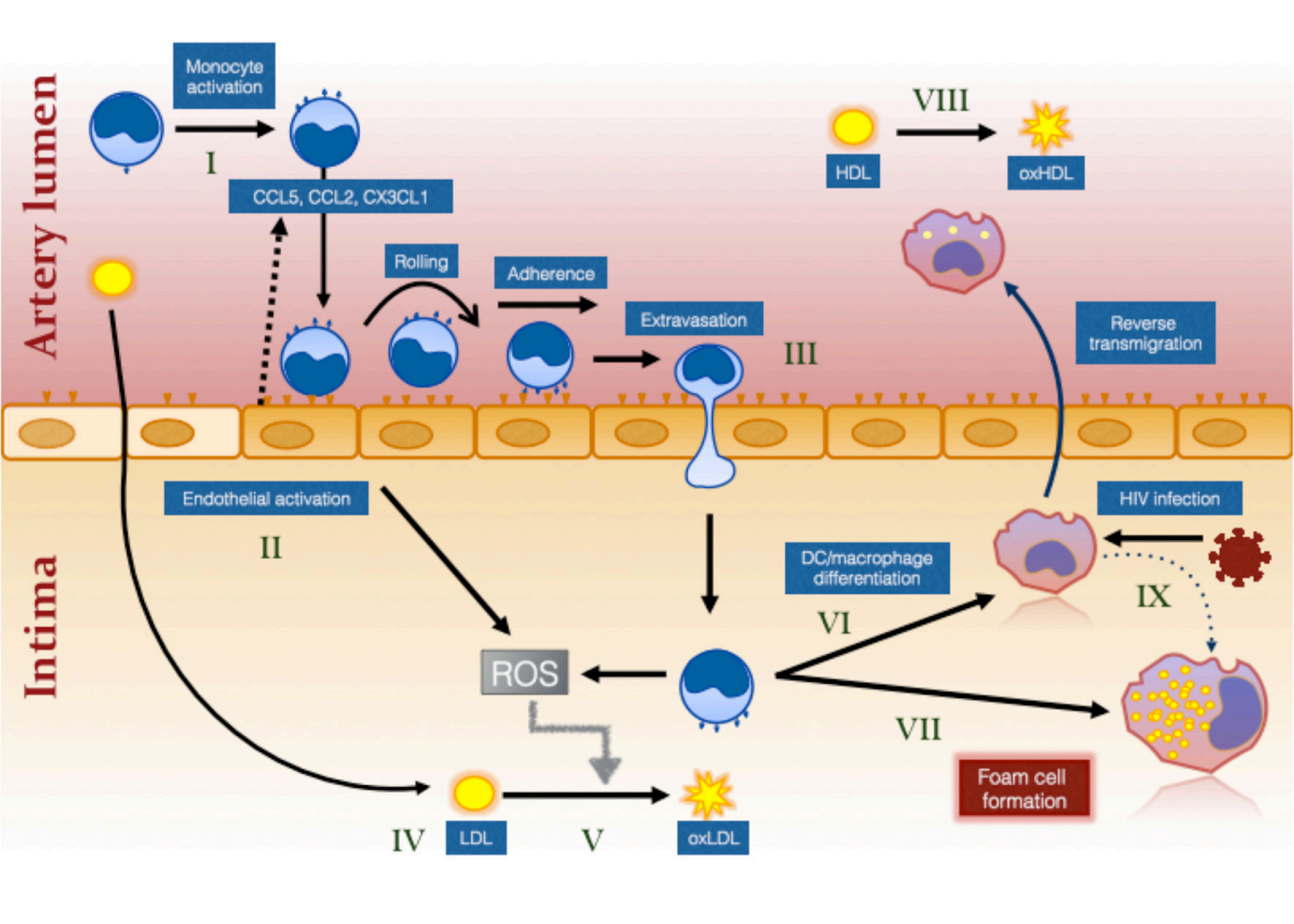
The effect of HIV infection on monocyte- and inflammation-mediated mechanisms of foam cell formation. HIV infection causes systemic monocyte activation (I) due to factors unresolved by ART including residual HIV viremia, CMV reactivation, elevated bacterial ligands and oxidative stress. Gut bacterial ligands (eg., lipopolysaccharide; LPS) activate classical and intermediate monocytes via CD14/TLR4 receptor while viral ligands activate non-classical monocytes via TLR7/8 (144). Unresolved inflammation also activates the endothelium (II) which secrete chemokines to attract different monocyte subsets (not illustrated) via specific chemokine receptors, CCR2, CCR5, and CX3CR1 (99). Intermediate monocytes in particular exhibit increased pro-atherogenic properties (127). Activation of the endothelium and of monocytes results in greater monocyte adherence, rolling, firm adhesion and extravasation, the last via either paracellular, or transcellular mechanisms (III) [reviewed in (145)]. Cholesterol accumulates in the intima due in part to ART-induced dyslipidemia and to increased traditional risk factors in PLWH (IV). This is oxidized by the activated myeloid and endothelial cells which produce reactive oxygen intermediates (ROS) (V). Monocytes that have migrated into the intima ingest LDL and oxLDL via LDL receptor and CD36/SR-A1/II (131), respectively and either mature into monocyte–derived dendritic cells (VI) and reverse migrate out of the intima or mature into immobile foam cells (VII). Changes in monocytes, including decreased ABCA1 expression (126), favors pathway (VII) over pathway (VI). Oxidation of HDL (VIII) that also occurs in virologically suppressed PLWH impairs the protective function of this lipoprotein and further increases the risk of atherosclerotic plaque formation. Direct infection of macrophages in the intima also induces an atherogenic phenotype promoting foam cell formation (IX).

Monocytes initiate the process of atherosclerotic plaque formation following their transmigration from blood into the intima of arteries at sites of activated endothelium and cholesterol deposition (“fatty streaks”) (Figure 1). Here they mature into foam cells which are macrophages containing large numbers of lipid droplets (94). They also have important inflammatory roles in atherosclerotic plaque progression and rupture. The present review focuses on their role in atherosclerotic plaque initiation.

Monocytes are currently classified into subsets based on the expression of CD14 which is the co-receptor for Toll-like receptor 4 (a pattern recognition receptor that recognizes lipopolysaccharide derived from gram-negative bacterial cell walls) and CD16 (the intermediate affinity IgG receptor, FcγRIII). The majority of monocytes in circulation are CD14^hi^CD16- classical monocytes which represent ~90% of the total in healthy individuals, while non-classical or patrolling monocytes (CD14^dim^CD16+) and intermediate monocytes (CD14^hi^CD16+) are minor subsets which each represent ~5% of circulating monocytes. It is likely that monocytes in circulation represent a continuum of classical monocytes newly emerging from the bone marrow and gradually maturing into intermediate then non-classical monocytes (95) or re-emerging from tissue sites such as the spleen (96, 97).

Monocyte subsets have different functional properties and must therefore be considered separately with respect to their roles in inflammation and atherosclerosis. Recruitment of monocytes to arteries at sites of inflammation is directed by chemokines released by activated endothelial cells, but the multiplicity of chemokines and their receptors makes it difficult to determine the role of individual chemokines. Mouse models have been particularly valuable in this context: using the ApoE knockout/western-type diet model of atherosclerosis, early experiments revealed the importance of the chemokine receptor CCR2 in atherogenesis (98) and subsequently that the murine equivalent of the classical monocyte subset (Ly6C^hi^ CCR2+ monocytes) migrates in response to the chemokine CCL2 (99, 100). It was reported by Tacke et al that classical monocytes also migrate into atherosclerotic plaques in response to the fractalkine receptor, CX3CR1 which is unexpected since they do not express high levels of this receptor, and furthermore that non-classical monocytes (Ly6C^lo^CX3CR1+) mainly migrate into atherosclerotic lesions in response to CCR5 but not CX3CR1 even though the latter is highly expressed in this subset (99). The recruited monocytes were shown in these studies to differentiate into lesional macrophages and therefore to potentially participate in disease progression. Neither study examined the migration of intermediate monocytes which are not a well-defined subset in mice. Support for the role of CCR5 in monocyte recruitment into atherosclerotic plaques has been obtained using the ApoE knockout mouse model of atherosclerosis, where it was shown that monocyte recruitment into plaques, and plaque progression, was reduced with treatment using the CCR5 antagonist Maraviroc, which is of relevance to PLWH who may be treated with ART regimens containing this drug (101). Following AMI, monocytes are also recruited into atherosclerotic plaques with distinct kinetics to co-ordinate left ventricular repair. In this setting, classical monocytes are recruited initially in response to CCR2 ligands, where they phagocytose necrotic debris and orchestrate pro-inflammatory responses (102). Non-classical monocytes are recruited later and participate in tissue repair, angiogenesis and extracellular matrix deposition, although in this study their migration was reported to be dependent on fractalkine receptor (*ibid*).

There are no extensive data on how HIV infection, especially in settings of virologic suppression with ART, affect chemokine receptor expression on individual monocytes to modulate monocyte recruitment to atherosclerotic lesions. It is reported that HIV-infection alters proportions of CCR2- and CX3CR1-expressing monocytes in circulation, but this may be due to alterations in the distribution of monocyte subsets. In a cohort of virologically suppressed HIV-positive individuals with low cardiovascular risk, we have previously demonstrated an association between the proportion of CX3CR1+ CD16+ monocytes and cIMT, supporting a potential link between monocyte recruitment and atherosclerosis (103). We, and others, have reported that intermediate and non-classical CD16+ monocytes, are expanded in viremic PLWH (104–108) but no difference in subset proportions is seen in PLWH who are virologically suppressed (109). Recently, using a well-controlled longitudinal cohort of PLWH commencing ART, we showed that intermediate and non-classical monocyte proportions decreased rapidly on commencement of ART, reaching control levels within ~6 months of therapy (108). Taken together, these data suggest that when assessing the roles of individual subsets in CVD risk and progression, the impact of HIV infection and of ART on monocyte subsets must be considered.

Recruitment of monocytes to sites of atherosclerotic plaque formation is favored by increased expression of adhesion receptors such as ICAM-1 on activated endothelial cells (110). Systemic inflammation may also activate circulating monocytes to increase expression of adhesion receptors such as CD11b/CD18 and CD11d/CD18 (111) which recognize their cognate ligands such as ICAM-1 and VCAM-1 expressed on activated endothelium (112). Following attachment, monocytes transmigrate across the endothelium and enter the intima where they ingest deposited lipids via scavenger receptors including CD36 and scavenger receptor A1 and AII, the major receptors for oxLDL expressed on macrophages. Monocytes can reverse transmigrate and transfer lipid to acceptor molecules such as HDL, a process thought to maintain the health of the artery and regress atherosclerotic plaque development (113, 114) or they can differentiate into foam cells, which are large, immobile cells (115) characterized by lipid accumulation in lipid droplets and lysosomes, and are among the earliest pathogenic feature of atherosclerotic plaque formation. Lipids derived from oxLDL accumulate in lysosomes and in this intracellular compartment, unlike lipids found in lipid droplets, do not readily participate in cholesterol efflux pathways (116). Lysosomal foam cell lipids persist as a stable store, as data from the White Carneau Pigeon model of atherosclerosis indicates, after a change in diet (117). Thus, the differentiation/maturation pathway of the monocyte, which is dictated in part by the type of lipid ingested, may determine the initiation of atherosclerotic plaque development.

While models of atherosclerotic plaque development rightly emphasize the role of lipid accumulation in the artery wall as the initial stimulus activating the endothelium, attracting monocytes and acting as a substrate for foam cell formation, it is also clear that inflammatory mechanisms intersect this mechanism by independently activating monocytes and endothelial cells and oxidizing lipids by promoting oxidative stress pathways (118) (See Figure 1). However, as discussed above, PLWH may have elevated risk of atherosclerosis independent of traditional risk factors such as elevated total cholesterol and LDL_c_, and this increased risk may in part be accounted for by their increased levels of inflammation and monocyte activation. It is important to realize that foam cell formation by monocyte-derived macrophages may occur in response to toll-like receptor ligands including HIV ssDNA (119), rather than lipoprotein particles [reviewed in (118)] which is of relevance to HIV infection where there is evidence for elevated levels of circulating endotoxin as well as other TLR ligands derived from microbial translocation even in virologically suppressed individuals (47, 120, 121). Finally, as discussed further below, foam cells may be induced following alterations in macrophage cholesterol metabolism and efflux and metabolic changes in circulating monocytes may predispose them to differentiate into foam cells following transendothelial migration.

## How Does HIV Infection Influence Monocytes to Promote CVD?

The association of plasma biomarkers of myeloid activation with CVD in HIV-positive individuals, especially where other markers of inflammation are not strongly associated (44, 53), suggest that activated monocytes or macrophages play a direct role in promoting atherosclerosis. However, while plasma levels of molecules released from activated myeloid cells such as sCD163 may be useful prognostic biomarkers, they do not provide information about the mechanistic link between CVD and particular myeloid cell types and their functions. For this, functional studies of cells obtained from PLWH are required.

Macrophages containing the major HIV antigen (p24 capsid protein) have been detected in coronary arteries present in autopsy material obtained from patients receiving ART (63). These cells were adjacent to the lipid core of the plaque and had morphological characteristics of foam cells. Using an *in vitro* infection model, Bukrinsky and colleagues showed that HIV infection of monocyte-derived macrophages impaired reverse cholesterol transport and down modulated the cholesterol transporter ATP Binding Cassette family member A type 1 (ABCA1) via a mechanism requiring the HIV accessory protein, Nef (122). This molecule is the major transporter through which cholesterol is effluxed from macrophages to acceptor molecules like apolipoprotein A1 in HDL particles. It was further shown that injecting Nef protein into mice to achieve circulating levels reported in viremic HIV+ individuals, exacerbated dyslipidemia and development of plaque in the high fat diet ApoE^−/−^ mouse model of atherosclerosis. This work suggests a plausible mechanism for promoting atherosclerosis in PLWH, but its significance to their CVD risk depends on the prevalence of HIV-infected macrophages in coronary arteries and on the concentration of Nef circulating in virologically suppressed individuals, for which there is currently a paucity of information. It would be of interest to use modern techniques such as DNA- and RNAscope to evaluate HIV infection in resected coronary arteries and to accurately determine circulating levels of Nef protein in a current cohort of PLWH.

Given the persistence of monocyte activation in ART-treated PLWH and its association with CVD, we reasoned that monocytes present in PLWH may have pro-atherogenic functional properties induced via bystander mechanisms dependent on HIV infection, even if these cells are not infected with the virus. To address this, we used a static model of atherosclerotic plaque development in which a type 1 collagen matrix is overlaid with a monolayer of primary human endothelial cells (HUVEC) and monocytes freshly isolated from study subjects are added (123, 124). In this model, monocytes transmigrate across the HUVEC monolayer to enter the collagen matrix, and the fate of the monocytes is followed over the subsequent 48 h. This model has been used extensively to determine the reverse transmigration properties of monocytes and the endothelial cell-expressed molecules such as PECAM-1 governing extravasation (113, 125), but had not previously been used to compare monocyte behavior from clinical cohorts. Using this model, we have shown that monocytes isolated from virologically suppressed PLWH have a higher propensity to mature into foam cells than monocytes from age-matched HIV-negative individuals, and that plasma from these individuals contains factors that promote this functional phenotype (126). In this study, monocytes from PLWH also exhibited impaired reverse transmigration out of the collagen and across the HUVEC monolayer, which is possibly linked to their greater propensity to mature into immobile foam cells. These functional defects were accompanied with impaired cholesterol efflux and reduced ABCA1 expression at the mRNA level. We are currently examining monocytes isolated from a larger group of virologically suppressed PLWH with low/medium FRS, to determine whether we can detect the same monocyte atherogenic phenotype and, if so, whether interventions to reduce CV risk will improve their functional properties (Angelovich, Hearps, Trevillyan, Hoy, and Jaworowski unpublished).

Experiments we have conducted using this *in vitro* model have shown that the intermediate monocyte subset has the greatest propensity of the three monocyte subsets to differentiate into foam cells (127) and expresses the highest levels of intracellular TNF and IL-6, both in the steady state and in response to LPS (128). Thus, to the extent that the proportion of this monocyte subset is elevated in PLWH, this may exacerbate the pro-atherogenic properties of monocytes in these individuals.

It is interesting that monocytes isolated from virologically suppressed PLWH possess similar functional defects in cholesterol transport as the HIV-infected monocyte-derived macrophages described by Bukrinsky and colleagues, although there is no evidence to date that Nef is the circulating factor causing this functional change in the subjects we studied. Monocytes from relatively old but healthy HIV-negative individuals have similar properties (127) strengthening the view that there are similarities in monocyte inflammatory behavior induced by HIV and by healthy aging, and suggesting that mechanisms apart from Nef contribute to this phenotype.

The model we use to study monocyte atherogenicity *in vitro* involves activation of the endothelial monolayer with TNF, simulating an inflammatory milieu *in vivo*. TNF can decrease expression of ABCA1 (129) and via this mechanism promote foam cell formation, but in the above experiments it was removed from the culture medium before monocytes were added. Furthermore, ABCA1 expression was decreased in freshly isolated monocytes from HIV-positive subjects. The factors that reduce ABCA1 expression in monocytes from PLWH *in vivo* remain to be established. As a static model, this system does not provide reliable information about the adhesion and transmigration properties of monocytes *ex vivo*, only their behavior post migration. Measurements conducted under shear flow conditions will need to be made to address whether monocytes from virologically suppressed PLWH have abnormal endothelial adhesion and transmigration properties.

## Abnormal Lipoproteins in PLWH may Influence Monocyte BEHAVIOR

It is important to consider that the pro-atherogenic phenotype of monocytes from virologically suppressed HIV-positive individuals revealed using the atherosclerotic plaque model is evident in the absence of added exogenous lipid or lipoprotein particles. However, we have used the same model to show that transmigrated monocytes can be induced to differentiate into foam cells by the addition of known atherogenic lipids such as oxLDL (124) and by LPS (127) into the culture medium. This may, in part, explain the pro-atherogenic properties of plasma from these individuals. LDL is oxidized (89) and taken up by macrophages via scavenger receptor A and CD36 (130–132) and since their expression is not regulated in a cholesterol-dependent manner, this promotes foam cell formation.

HDL is normally a protective lipoprotein with respect to CVD since its major protein component, apolipoprotein A1, functions as a cholesterol acceptor from cells. However, HDL in plasma of PLWH exhibits an increased level of oxidation, and the levels of oxidized HDL (oxHDL) correlate with markers of systemic inflammation such as IL-6 and hsCRP (133). We have isolated HDL particles from plasma obtained from a limited number of virologically suppressed PLWH, incorporated the isolated particles into the atherosclerotic plaque model and showed that they promote foam cell formation by monocytes from healthy, HIV-negative individuals. This behavior was associated with defective redox properties of the HDL particles (134). Functional defects in HDL particles isolated from PLWH have been reported by others, specifically a decreased level of the enzymes Paraoxonase (PON) 1 and 3 and a decreased level of PON redox activity (135). PON enzymes have important anti-inflammatory properties: in the context of atherosclerosis they inhibit the oxidation of LDL particles (136–138) and reduce monocyte-attracting chemokine expression by the endothelium (139, 140). PON also inhibits cholesterol biosynthesis (141) and stimulates cholesterol efflux from macrophages (89), properties that reduce the propensity of macrophages to develop into foam cells. While human macrophages do not express PON1 or PON 3 these effects can be mediated by PON delivered via HDL particles (*ibid*).

The behavior of monocytes isolated from virologically suppressed PLWH and of monocytes from healthy subjects in response to HDL particles from PLWH are consistent with the observation of lower PON1 and 3 levels in these particles. As PON can be displaced from HDL particles by inflammatory proteins such as serum amyloid A in circumstances such as acute infection (142, 143) it will be critical to understand which factors promote loss of PON1 and 3 in chronic HIV infection.

## Conclusions

While the relative risk of death from AMI has decreased significantly for PLWH and has approached that for comparable members of the general community, CVD will become an increasing cause of morbidity and mortality as the HIV-positive population ages. It is still unclear to what extent the risk of atherosclerosis is elevated in patients who commence ART at high CD4 counts, especially as individuals may be treated with ART for many decades, which is longer than follow up studies conducted to date. Monocytes are principal effectors that initiate atherosclerotic plaque formation at sites of endothelial activation and damage. The “decision” of monocytes to differentiate into foam cells rather than extravasate from the intima influences the chance that initiation and progression of atherosclerotic plaques occurs. Functional studies using freshly isolated monocytes from virologically suppressed HIV-positive individuals are consistent with a phenotype that promotes atherosclerosis and therefore suggest that therapies designed to target this phenotype will prove beneficial. Future studies should assess the impact of therapies that reduce inflammation including statins and agents that target pathogen-induced monocyte/macrophage activation to reduce the tendency of monocytes to migrate across activated endothelia and differentiate into foam cells. A better understanding of the transcriptional changes in monocytes in PLWH, especially those involved in cholesterol/lipid metabolism and accumulation, will inform pathways that need to be targeted to prevent this. Emerging technologies such as improvements to [^18^F] FDG-PET might be useful in assessing how successful interventions are at reducing the accumulation of activated macrophages in the heart. PLWH should be monitored for CV risk, encouraged to reduce modifiable risk factors and treated appropriately. The use of novel biomarkers such as oxLDL and oxHDL to improve risk prediction in the setting of HIV infection should also be considered.

## Author Contributions

AJ conceived and wrote the manuscript. AH, JH, and TA contributed to the manuscript. All authors reviewed and approved the final manuscript.

### Conflict of Interest Statement

The authors declare that the research was conducted in the absence of any commercial or financial relationships that could be construed as a potential conflict of interest.
